# Imagining machine vision: Four visual registers from the Chinese AI industry

**DOI:** 10.1007/s00146-023-01733-x

**Published:** 2023-08-01

**Authors:** Gabriele de Seta, Anya Shchetvina

**Affiliations:** 1 Department of Linguistic, Literary and Aesthetic Studies, University of Bergen, Bergen, Norway; 2 GRK „Kleine Formen“, Humboldt University, Berlin, Germany

**Keywords:** Artificial intelligence, China, Machine vision, Sociotechnical imaginaries, Tech industry, Visual culture

## Abstract

Machine vision is one of the main applications of artificial intelligence. In China, the machine vision industry makes up more than a third of the national AI market, and technologies like face recognition, object tracking and automated driving play a central role in surveillance systems and social governance projects relying on the large-scale collection and processing of sensor data. Like other novel articulations of technology and society, machine vision is defined, developed and explained by different actors through the work of imagination. In this article, we draw on the concept of sociotechnical imaginaries to understand how Chinese companies represent machine vision. Through a qualitative multimodal analysis of the corporate websites of leading industry players, we identify a cohesive sociotechnical imaginary of machine vision, and explain how four distinct visual registers contribute to its articulation. These four registers, which we call *computational abstraction*, *human–machine coordination*, *smooth everyday*, and *dashboard realism*, allow Chinese tech companies to articulate their global ambitions and competitiveness through narrow and opaque representations of machine vision technologies.

## New ways to see our world

The camera zooms into Earth from space, through downtown Shanghai, into a Chinese couple’s futuristic bedroom. As the couple wakes up, they mirror themselves in an augmented reality screen and enjoy a coffee while logging into their personalized recommendations with a face scan. An English-language voiceover proclaims:

Have you ever thought about it? Where is the future? One day, when we wake up, like the first sunshine in the morning, the future has shined into the reality [*sic*]. CloudWalk, from the beginning of every day is making changes to our smart life. […] With continuous investment in technology R&D, research and development such as face recognition, crowd counting, person reidentification, and optical character recognition, it has deeply cultivated in many fields, such as finance, security, transportation, education, and so on, helping all aspects of technology life, consolidating the foundations for our smart future, working together for the harmonious development of society.

This scene happens in a promotional video featured on the website of CloudWalk, a Chinese machine vision company which describes itself as “the largest AI supplier in the financial industry”.^1^ The video continues with the man driving an automated car to his workplace and the woman going jogging under the watchful eyes of posture analysis software.

Other Chinese tech companies represent machine vision through more concrete visualizations. In contrast to the futuristic imagery chosen by CloudWalk, an advertising video for Huawei’s HoloSense cameras focuses on both the hardware components and the computation happening behind the lens: a black and grey minimalist rendering of the camera compares its design to a droplet of water; as the soundtrack shifts from ominous ambient beats to an aggressive guitar riff, the product spins and explodes in a rendering of its internal components, with abstract threads of luminous data illustrating how the surveillance camera is connected to larger networked systems. Some product videos also offer a glimpse of the world as seen from the machine’s point of view. Hikvision, another leading surveillance system manufacturer, advertises a line of thermal cameras through a promotional video including footage of people and objects in the false colors of the infrared spectrum, commented by a British-accented voiceover:

Hikvision Thermal Cameras see the world in a new way. Mountains and streams, deserts and seas. There is more to the Earth that we can see. At Hikvision we always believe there are new ways to see our world.

All the aesthetics spanned by these clips – futuristic urban spaces, convenient interactions with automated systems, disembodied flows of data and dashboard renderings – come together in the promotional video for Wanxiang (‘Ten Thousand Images’), a “comprehensive city governance software platform” developed by Chinese artificial intelligence company Megvii. Surveillance feeds and city management dashboards are displayed on massive screens in a police control room; a glowing wireframe rendering of a surveillance camera exemplifies how data flows from light sensors to cloud computing servers; glowing dots on a dark background coalesce into the shape of a human brain to symbolize how data is collected and analyzed; eventually, the setting shifts back to an urban street scene, zooming into a pair of bullet cameras mounted on a lamp pole, which identify bikes parked in the wrong spot and warn passersby caught littering.

**Fig. 1:**
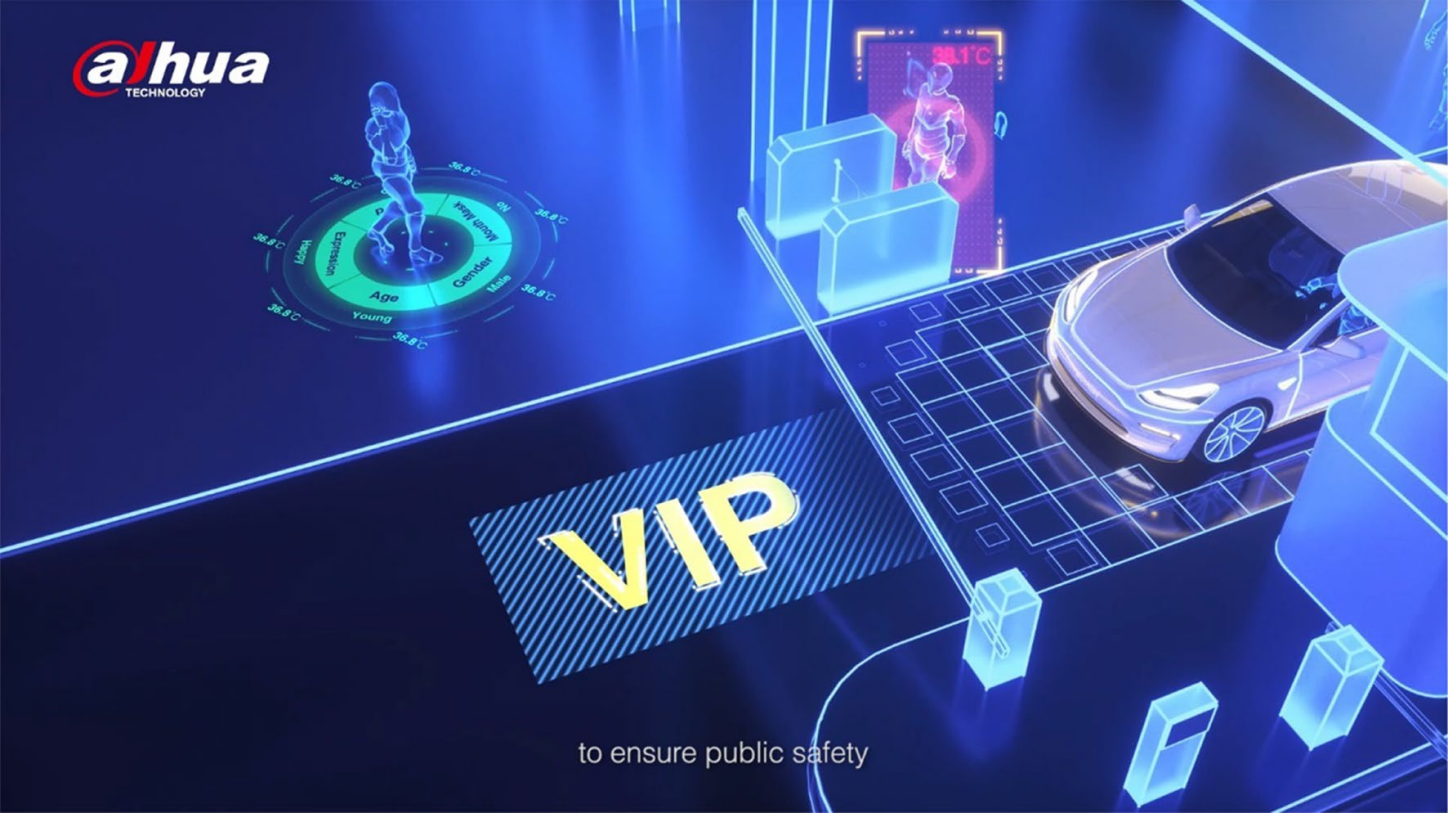
Still from a promotional video found on the Dahua website showcasing various machine vision applications (human attribute recognition, identity verification, temperature measurement, access control, vehicle tracking) through the common aesthetic style of wireframe 3D renderings in glowing blue hues

These examples of promotional videos sourced from the websites of various Chinese tech companies offer a representative overview of how machine vision is represented by leading industry actors [Fig. 1]. Machine vision, broadly intended as the application of computational techniques to derive information from visual inputs, is one of the main applications of machine learning. In China, where industry hype around big data and artificial intelligence has driven a decade of research and development, machine vision occupies more than a third of the industry market (Tencent Research Institute 2020). Machine vision applications such as facial recognition, object tracking and autonomous vehicles are amongst the most visible manifestation artificial intelligence in national public discourse, which is dominated by industry actors depicting it with “over-hyped and economy-focused” coverage (Zeng et al. 2022, 332). As they intersect with other industry buzzwords like big data and smart city, machine vision technologies play a central role in national surveillance systems and social governance projects relying on the large-scale collection and processing of visual data (de Seta 2020). While these technologies are often correlated to the growth of China’s artificial intelligence industry during the mid-2010s, machine vision has been entangled with surveillance and security industries since at least the early 2000s, when governmental projects like the Golden Shield were being established (Walton 2001). From an industry reliant on foreign investment and imports, machine vision has become one of China’s global exports (Weber and Ververis 2021), and since a 2013 push for the national procurement of security hardware, several tech companies like CloudWalk, Huawei, Hikvision and Megvii have emerged as industry leaders (Huang and Tsai 2022).^2^

In this article, we examine how leading Chinese tech companies imagine machine vision. We do so by analyzing a specific genre of multimedia text–the corporate website–through the concept of sociotechnical imaginary. By analyzing how the major players in China’s artificial intelligence industry represent machine vision on their websites, we aim at demonstrating that corporate actors rely on a limited number of visual registers to articulate a cohesive sociotechnical imaginary that in turn becomes dominant in both public debate and popular representations of these technologies. In the first section of the article, we summarize theoretical discussions of sociotechnical imaginaries and make a case for the need to analyze the ones articulated by corporate actors through different visual registers. In the note on method, we detail our research design, data collection and analysis, explaining how corporate websites can become rich sources for the analysis of industry imaginaries. The central part of the article presents our research results, which are divided in two sections: one presenting general observations on China’s machine vision imaginary, and another detailing the four visual registers emerging from our data. In the conclusion, we summarize how visual registers contribute to the articulation of a cohesive sociotechnical imaginary of machine vision, and offer several takeaways on China’s machine vision industry as well as directions for future research on the topic.

## Sociotechnical imaginaries and visual registers

As evidenced by the promotional videos described in the previous section, imagination is an important resource for companies seeking to illustrate and promote products based on machine vision technologies. Given the technical complexity and variety of machine vision applications–which include QR code scanners, biometric access points, image analysis services, surveillance cameras, automated vehicles, and more–it is not surprising that industry actors rely on simplifications, exaggerated claims and science-fictional tropes to market their offerings. Through this sort of representations, companies imagine machine vision and, in turn, shape how machine vision is imagined by the public: hence, the importance of attending to how the imaginaries of machine vision are constructed. Imagination has been a prolific domain for interdisciplinary inquiry (Strauss 2006), as exemplified by key contributions like Taylor’s theorization of “modern social imaginaries” (2004), Marcus’s collection of “technoscientific imaginaries” (1995) and Flichy’s charting of the “internet imaginaire” (2007). In these works, the notion of imaginary functions as a more nuanced alternative to related concepts like myth, belief, fantasy, utopia or ideology, while also indicating something broader than an individual vision and more scalable than a societal project.

The concept of “sociotechnical imaginaries”, originally proposed by Sheila Jasanoff and Sang-Hyun Kim to understand “collectively imagined forms of social life and social order reflected in the design and fulfillment of nation-specific of scientific and/or technological projects” (2009, 120), has been expanded by the same authors to include a broad range of “powerful cultural resources that help shape social responses to innovation” (2013, 190). This concept has proven to be extremely relevant for scholarly inquiry into a wide variety of subjects, particularly from STS perspectives, where it has been articulated in pluralized and polyvocal ways (McNeil et al. 2017). Analyses of sociotechnical imaginaries often follow the ebbs and flows of technological hype cycles by tracking, for example, transformations in consumer data mining (Turow et al. 2015), promissory narratives of the smart city (Mertia 2017; Sadowski and Bendor 2019), patterns in the myth-making about artificial intelligence (Natale and Ballatore 2020), infrastructural restructurings of digital payments (Mützel 2021), or the imagined affordances of robotic touch (Barker and Jewitt 2022). Discovering the sociotechnical imaginaries of countless innovations, industries and practices leads to a multiplication of specialized versions of this concept, including the surveillance imaginary (Lyon 2017), the algorithmic imaginary (Bucher 2017), the data analytic imaginary (Beer 2018), and many more.

Besides existing in a multitude of variations and degrees of specificity, sociotechnical imaginaries are, most importantly, *articulated*. Patrice Flichy noted this early on when he argued that technological imagination always relies on “an articulation between utopia and ideology” (2007, 11). Even the most powerful sociotechnical imaginaries, as they oscillate between utopia and ideology, are not necessarily embraced without friction: Internet users, for example, both naturalize and contest the global internet imaginary through narrative practices (Felt 2015, 192). Other actors also compete to articulate sociotechnical imaginaries. Narratives promoted by industries for marketing purposes find their way first into public debate and then into political discourse (Rieder 2018, 90). At the same time, entrenched myths determine how popular media “often, albeit inadvertently, reinforces a blurring of the line between fantasy and reality” (Elish and boyd 2018, 62). Similarly, works of art and narrative substantiate hopes and fears around novel technologies, influencing industry developers, public attitudes, and government regulations (Cave and Dihal 2019, 74). News media also take an active part in this process by being one of the main arenas in which articulation takes place (Vicente and Dias-Trindade 2021, 720). These complex loops result in surprising similarities between sociotechnical imaginaries across industries and national contexts (Bareis and Katzenbach 2021, 2–3), as well as in peculiarly localized and bounded articulations (Smith and Tidwell 2016).

As theorized by Tadeusz Józef Rudek in his overview of energy studies, the articulation of sociotechnical imaginaries develops across different scales: after emerging from private, individual visions, they become institutionalized, crystallizing into dominant and alternative ones, which in turn engender different expectations for future investment, policies and practices (2022, 232). In this article, we focus on the sociotechnical imaginary articulated by Chinese machine vision companies. Industry actors influence governance, representation and practices by projecting “promises, ideals and values” (Beer 2017, 20) onto their products. Cohesive and powerful “corporate sociotechnical imaginaries” offer enticing pathways to utopian futures passing through specific configurations of technology and sociality (Haupt 2021, 253); the hypes promoted by private companies function as “hot air” (Hockenhull and Cohn 2021) giving buoyancy to these visions and facilitating their translation into practice. Compared with the largely textual domains of policy and public discourse, industry imaginaries are often articulated through visual media, particularly as these are used in promotional materials. Research on sociotechnical imaginaries has recently shifted toward “aesthetics, values, and emotions” and their role in “storytelling, imaging, and imagining” (McNeil et al. 2017, 457); our study follows this shift, honing onto corporate visual aesthetics and their role in imagining machine vision.

In order to unpack the sociotechnical imaginary articulated by Chinese machine vision companies, we approach it as a composite of different representational styles which we call “visual registers”. The concept of register is drawn from linguistics, where it is commonly used to identify a repertoire of speech forms developed through reflexive social and historical processes (Agha 2004, 24–25). Linguistic registers span broad distinctions between formal and informal writing styles and more nuanced characterizations of speech choices among professionals or intimate partners. Registers are not limited to the analysis of language, but can also be applied to the study of material signifiers like commodities (Agha 2011) or of multimodal forms of communication like global consumer aesthetics (Jaworski 2015). Our definition of visual registers–distinctive combinations of aesthetic resources that are consistent across a communicative setting–follows recent research in social semiotics addressing the visual construction of institutions (Jancsary et al. 2017) or the visual communication of authenticity on social media (Blasch 2021). In the context of corporate websites, we understand visual registers to be consistent repertoires of aesthetic resources encompassing a broad spectrum of modalities ranging from basic design elements like backgrounds and fonts to complex multimodal objects like interactive demos and videos. The following section details how visual registers can be identified and correlated to a cohesive sociotechnical imaginary.

## Multimodal clickthrough: A note on method

This article seeks to answer two questions: what kind of sociotechnical imaginary do Chinese machine vision companies articulate, and how do different visual registers contribute to it? Among many potential data sources, we focus on official company websites. Given the rich and heterogenous contents of these websites (text, images, videos, interactive elements, etc.), we decided to collect enough data to support a qualitative multimodal analysis (Pauwels 2012). Following research projects of a similar scope and scale (for example Beer 2018), we selected a sample of companies by cross-referencing various lists ranking the fastest growing, most valuable or market-leading Chinese tech companies that offered machine vision products or self-identified as machine vision companies, and only kept the ones recurring in at least two of these lists [see Appendix 2]. This process resulted in thirteen companies, which we narrowed down to twelve during the data collection process since one of them (iFlytek) had no relevant machine vision products on offer. Our sample included very different companies, ranging from tech giants like Alibaba or Tencent which offer machine vision services as part of their cloud computing business, through artificial intelligence companies specializing in machine vision software products, to manufacturers of surveillance cameras, smart city systems, and drones. Table 1 lists the companies selected as our sample, which we roughly divided into three categories as the data collection progressed.

**Table 1:** Our sample of twelve Chinese tech companies offering machine vision products, divided in three broad industry categories

Tech giants	AI companies	Hardware manufacturers
Alibaba	CloudWalk	Dahua
Baidu	Megvii	DJI
ByteDance	SenseTime	Hikvision
Tencent	Yitu	Huawei

Data was collected by the authors between November 2021 and June 2022, following a process developed through a pilot study. This process, which we term *multimodal clickthrough*, is inspired by the walkthrough method proposed by Ben Light, Jean Burgess and Stefanie Duguay for the systematic, user-centered and critical study of apps (2018). Rather than tapping through app interfaces, we adapted this approach to the navigation of corporate websites–following links, playing videos, interacting with tech demos, exploring API documentation, and occasionally looking at source code. While clicking our way through websites, we collected two kinds of data: visual content (layouts, logos, icons, images, banners, animations, videos, etc.), and descriptive notes (general impressions, thematic coding, and close readings of relevant pages). This data was combined in multimedia documents, one for each company, with the aim of preserving the epistemic value of visual design resources and maintaining the website as a unit for further multimodal analysis (Pflaeging and Stöckl 2021). Since most of these companies had websites available in multiple languages, we decided to conduct clickthroughs of both the International English version and the Simplified Chinese one, gleaning important details about industry localization and self-presentation. To avoid potential loss of access to important information, websites were archived with a web preservation tool. Eight months of data collection resulted in twelve documents summing up to 170 pages of textual notes and upwards of 300 images and screenshots, which were then consolidated for a comparative analysis grounded in multimodal social semiotics (Kress and van Leeuwen 2001).

Both authors developed concepts and themes iteratively throughout the data collection; when analyzing the data, we focused on extracting general conclusions on the sociotechnical imaginary of machine vision and defining the characteristics of specific visual registers. This was done by coding the multimodal data qualitatively in search of repetitions, recurring semiotic resources, similarities and differences, as well as missing elements, which resulted in a number of emerging themes (Ryan and Bernard 2003). Guided by the principle of multimodality, our analysis was not limited to coding text and images, but also addressed their interplay in terms of framing, tone, rhetoric, and layout (Pauwels 2005, 610), which further consolidated thematic clusters. The self-reflexive fieldnotes included in each multimodal clickthrough allowed us to account for the fluidity of websites (temporal changes, responsive elements and interactive sections), while screenshots and saved images helped us analyze choices made about their visual design and spatial organization (Djonov and Knox 2014), tying themes to a historical moment and a local context. Finally, the concept of visual registers helped us correlate these themes to specific combinations of content, embodied positions, and compositional boundaries (Jancsary et al. 2017, 95). Through this qualitative analytical process, we were able to identify emerging themes in multimodal data and distill them into four visual registers that contribute to the articulation of a cohesive machine vision imaginary. The following sections detail the general features of this sociotechnical imaginary and explain how different visual registers contribute to it.

## China’s machine vision imaginary: Global, blue, competitive

In order to frame the general observations presented in this section, it is important to emphasize that the websites we analyzed belong to a very specific genre of multimedia text situated in a peculiar historical and geopolitical context: corporate self-presentation of China’s AI industry in the early 2020s. The twelve companies we sampled have different backgrounds – some are established players in China’s tech industry since the late 1980s or 1990s (Huawei, Alibaba, Tencent), while others are startup unicorns founded in the 2010s (ByteDance, CloudWalk, DJI). These companies also share commonalities and differences between categories. The four tech giants have only recently added machine vision products to their cloud computing platforms as part of an industry-wide pivot to artificial intelligence research and development; the four AI companies, conversely, focus squarely on machine learning applications, with machine vision systems often being the most prominent; hardware manufacturers embed machine vision in products like surveillance cameras, access terminals, smartphones and drones, and some of them are expanding into cloud computing. For all these companies, official websites are primarily marketing materials: on a surface level, they are designed to sell products to their target audiences; at the same time, as their cohesive aesthetics demonstrate, they construct and reinforce a specific imagination of machine vision, which in turn shapes how these technologies are understood (Beer 2018, 469). These websites and their striking resemblance also need to be examined in the context of Chinese web design, where a corporate penchant for copycatting competitors has led to overcomplicated and information-heavy pages, and where minimalism is perceived as sign of professionalism and high-end brands (Schaefer 2014).

**Fig. 2:**
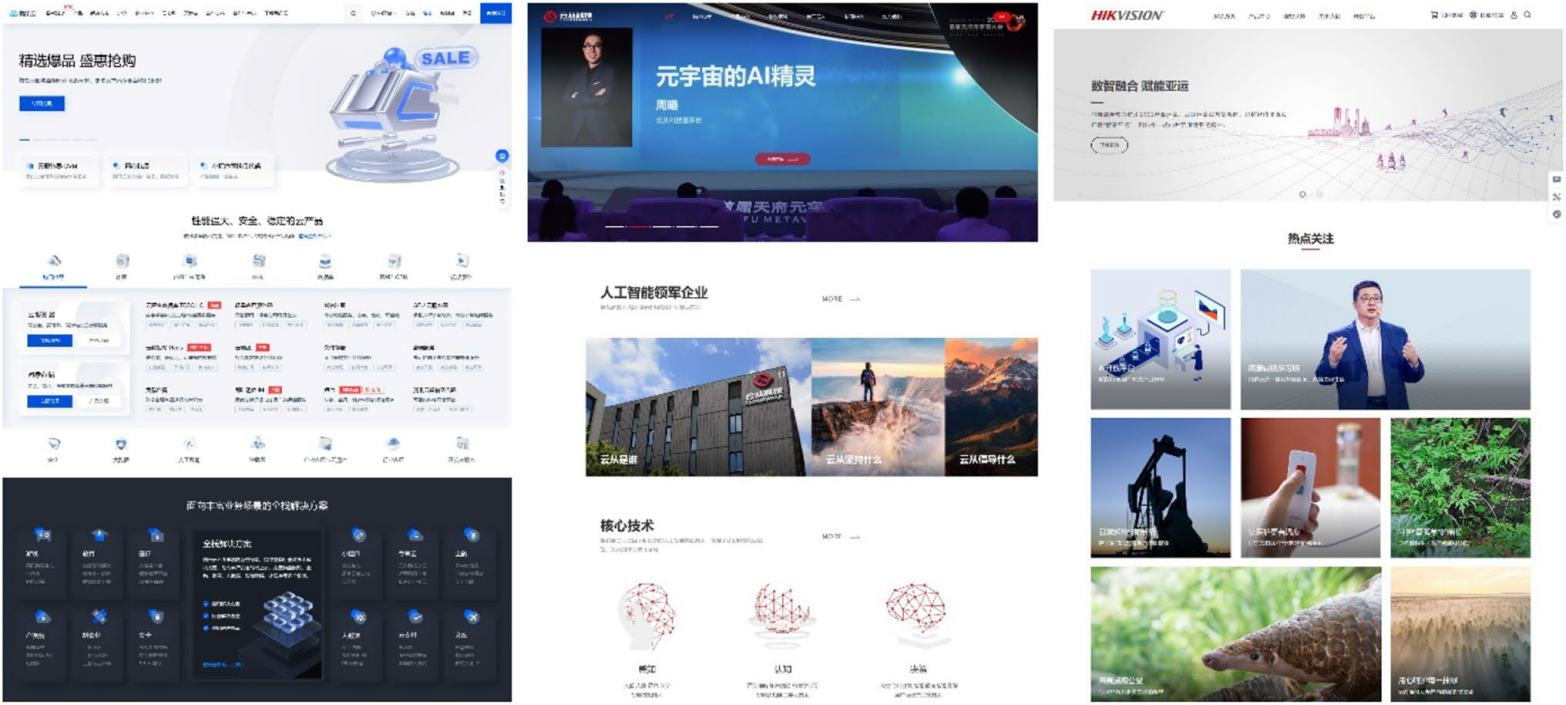
The top sections of three company homepages belonging to different categories: a tech giant (Tencent Cloud), an AI company (CloudWalk), and a hardware manufacturer (Hikvision), each showcasing the paradigmatic design choices of its category, as well as more general layout similarities

**Fig. 3:**
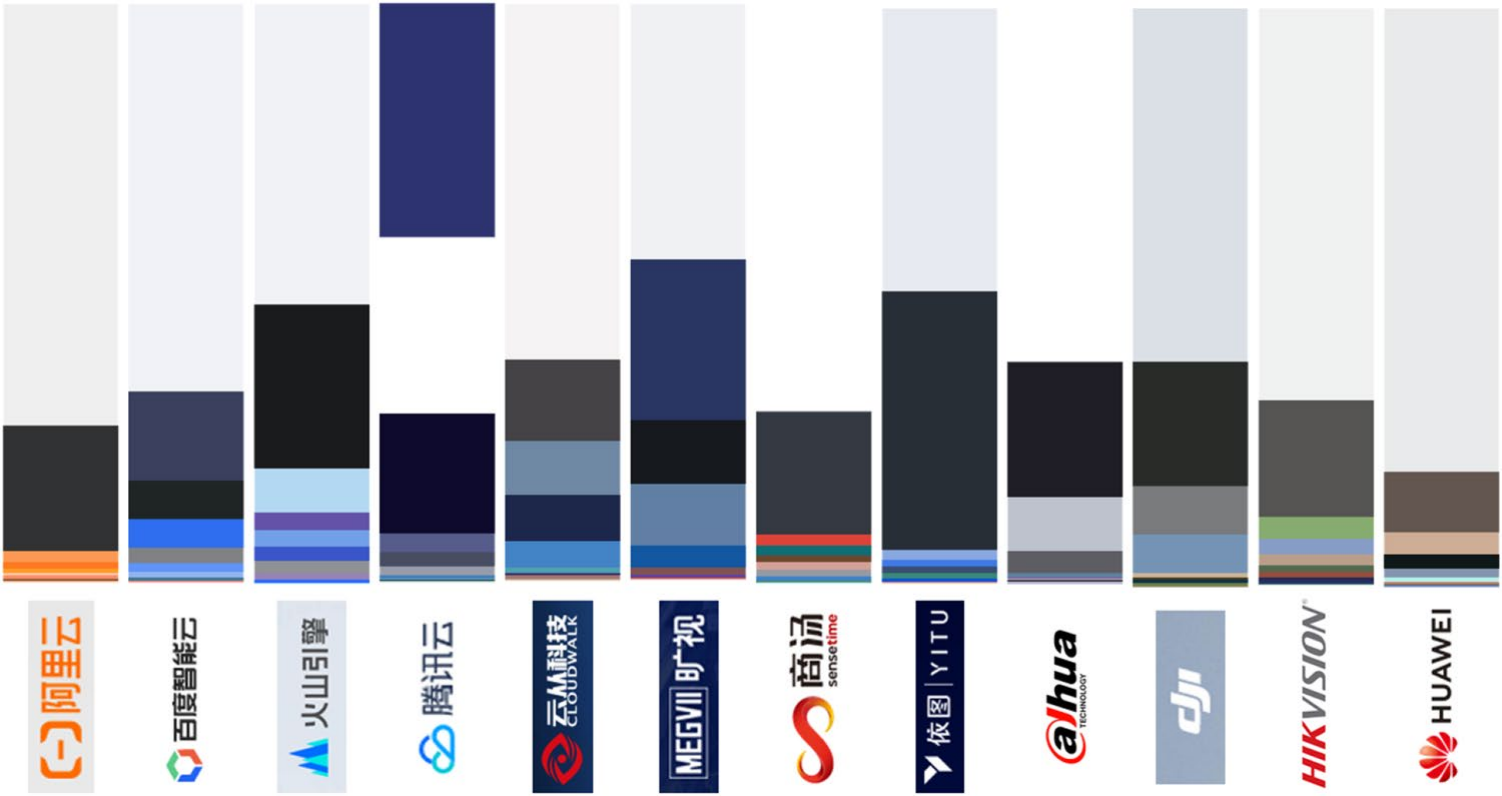
A comparison of proportional color palettes extracted from the homepages of each of the twelve companies in our sample (in order: Alibaba Cloud, Baidu Cloud, ByteDance’s Volcano Engine, Tencent Coud, CloudWalk, Megvii, SenseTime, Yitu, Dahua, DJI, Hikvision, Huawei). Created with TinEye’s MulticolorEngine

Generally speaking, all the twelve company websites share clear design choices and feature the markers of a specific global design period characterized by flat, minimalist and responsive design with lots of white space, scrolling-oriented navigation, and interactive elements (Chen 2017). The company logo is invariably positioned on the top left, embedded in a menu bar which usually links to “Products”, “Technologies”, “Solutions”, and “About Us” sections; right under it, banner-sized slideshows welcome visitors with splash images and bits of announcements or information about new products. Pages are structured around top-to-bottom scrolling, with the information conveyed by blocks of text accompanied by icons, infographics, and grids of photos [Fig. 2]. The dominant colors are various hues of blue on white and light grey backgrounds,^3^ with the accent color of each company (in many cases also a shade of blue) differentiating them [Fig. 3]. Our sample of companies also share the versioning of their websites into different languages: a national version in Simplified Chinese and an international version in English are the baseline, but most companies offer versions in several other languages; ByteDance is the outlier, having not yet published an international version of its cloud computing business website. Overall, the Chinese versions have more recent updates and are often slightly richer in content^4^–here, the DJI website is the exception, with both versions being identical in content and design. This consistency in contemporary design choices reflects the global outlook of the machine vision industry and projects a cohesive sociotechnical imaginary. At the same time, the uniformity allows minor details to become relevant through comparison, and stylistic patterns exemplify how major industry players position themselves in specific market segments.

One of the first results gleaned from our comparative analysis was the realization that specific web design choices map quite accurately to the three categories in which we had divided the twelve companies: tech giants, AI companies and hardware manufactures. For example, all four tech giants position their machine vision products (mainly software ones) in their cloud computing business website (i.e., Alibaba Cloud, Baidu Cloud, Tencent Cloud, and ByteDance’s Volcano Engine). The homepages of these four websites are nearly identical in structure and content, with a rather long footprint, landing banners featuring CGI representations of computation, no photos, lines of minimalist icons earmarking the main features of their products, and interactive tables with service pricing comparisons. Similarly, artificial intelligence companies share a preference for shorter and less information-heavy homepages, with taller landing slideshows, grids of photos with very brief textual captions, and stylized icons representing computational processes. The same is true for the homepages of hardware manufacturers, which feature even less content, usually limited to a banner-sized slideshow on the top, a mosaic of photos of products and scenarios, a selection of news, and icons linking to product categories. Shifts in the industry prove that this observation is consistent and not coincidental: hardware manufacturers like Hikvision or Huawei, which recently branched into the cloud computing business, have launched separate websites which follow the design choices of the other category. This demonstrates how aesthetics are paramount in the articulation of a sociotechnical imaginary: subtle differences in the use of different visual registers are enough to signify industry shifts and product categories.

In terms of rhetorical framing, we noted that regardless of how central machine vision is for these companies, it is rarely explicitly mentioned or discussed on their websites. For example, Baidu Cloud presents itself as a cloud-based platform provider of artificial intelligence services. Tencent’s AI Lab lists machine vision among its four key research areas and even offers a definition (“Computer vision enables computers to understand the real visual world”), but the Tencent Cloud website bundles machine vision products with speech recognition and natural language processing under the broader category of artificial intelligence. ByteDance’s Volcano Engine website mentions *jiqi shijue* [machine vision] as the core technology behind several of its products, but promotes “groundbreaking algorithms” as its competitive edge. Similarly, Yitu’s website introduces the company’s mission to “explore the AI world” through its “world class algorithms”, and Megvii describes itself as.

a global leader in AI products and solutions. Our core competency is deep learning, a key driver of the AI revolution. We focus on areas in which algorithms can create critical value […]. We provide customers with full-stack solutions that integrate algorithms, software and hardware.

**Fig. 4:**
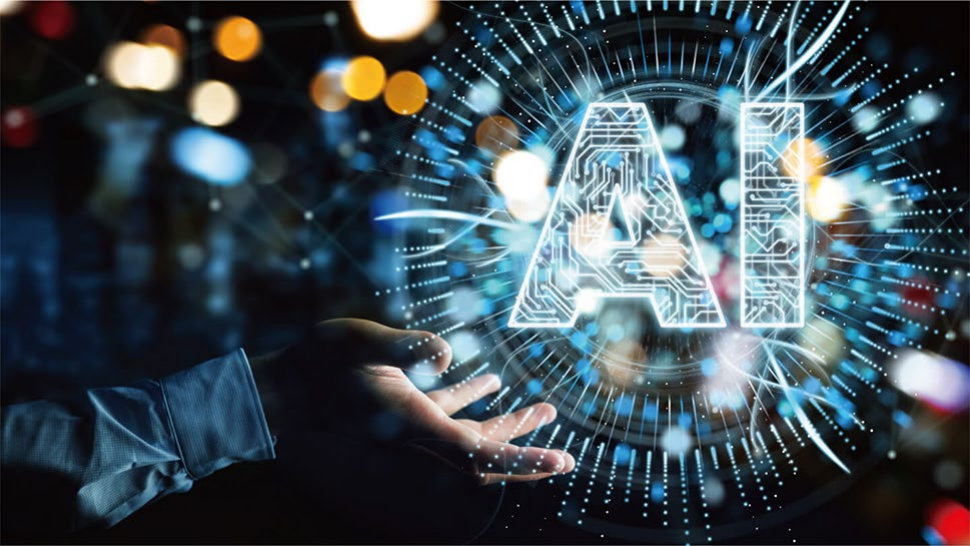
Literal representation of artificial intelligence used on the Hikvision website to illustrate deep learning technology, combining a stock image of an outstretched hand, a floating hologram of the “AI” acronym with circuit board pattern inside a ring of glowing blue data points

Hardware manufacturers like Huawei mention machine vision as feature of some of their products, obtained by combining artificial intelligence with their camera hardware through “advanced AI chips, able to load different algorithms”. The Hikvision website offers the most comprehensive buzzword soup: “advanced machine vision AI algorithms”. Across all these companies, terms like artificial intelligence, deep learning and algorithms take rhetorical precedence in the articulation of a machine vision imaginary. This is evidenced by one of the most recurring visual design elements: the letters “AI”, often glowing blue, stamped on microprocessors or projected as floating holograms [Fig. 4]. Emerging as a global, competitive and blue-tinted amalgamation of artificial intelligence, machine learning and algorithms, machine vision is most often presented as the applied result of abstracted computational processes, resulting in a variety of products that are marketable as “solutions” to practical needs (identifying people, scanning documents, guiding vehicles, etc.) or for vague future scenarios.

## Four visual registers

General comparative observations are useful for establishing that Chinese machine vision company websites articulate a cohesive sociotechnical imaginary. This section focuses on *how* different visual registers contribute to the machine vision imaginary. During our pilot study, we started coding different visual tropes related to the representation of machine vision and technology at large. These included, for example, the recurrence of holographic interfaces, the stylized motifs of glowing networks and data flows, the reliance upon stock photos of urban life and everyday activities, and the use of actual data visualizations. By correlating these tropes to broader themes emerging from the data (such as surveillance, accuracy, convenience, etc.) we identified three visual registers, which we expanded to four during the data collection: *computational abstraction*; *human–machine coordination*; *smooth everyday*; and *dashboard realism*. These registers encompass most of the visual elements of the analyzed websites, and as such offer a comprehensive mapping of the machine vision imaginary articulated by leading Chinese industry actors. The following subsections describe each visual register in detail, illustrating their features through examples from our data and connecting them to specific aspects of the machine vision imaginary.

### Computational abstraction

**Fig. 5:**
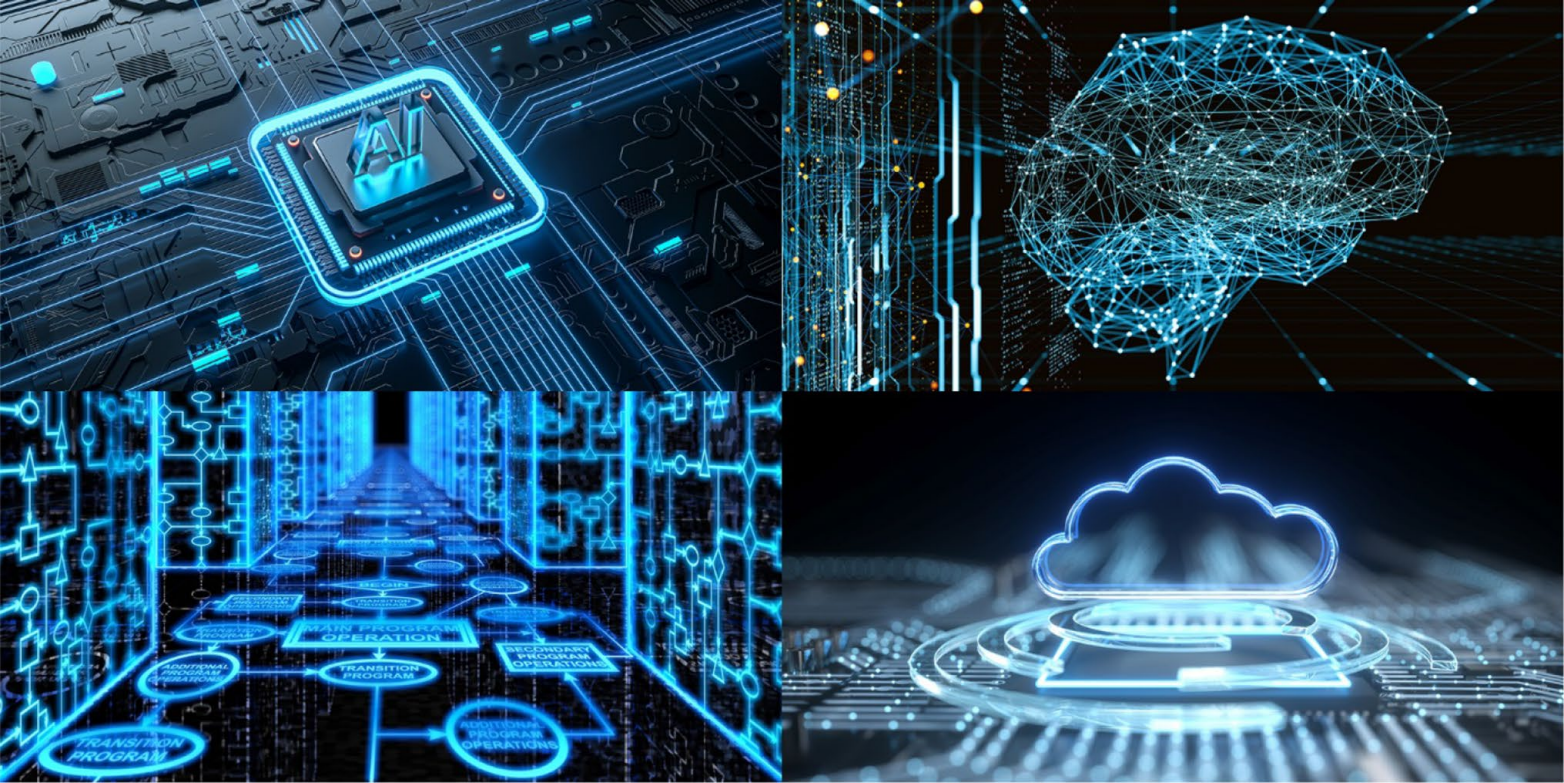
Some examples of computational abstraction–AI chips, circuit boards, networks, brains, algorithmic flowcharts, logic gates, clouds, and concentric rings–used on the SenseTime website to illustrate its Decision Intelligence core technology

The first visual register encompasses abstract representations of computation. While tangible products such as hardware devices or software systems can be easily represented through photographs and interface screenshots, computational processes happening inside chips and distributed between data centers are much more complex to illustrate. Machine vision companies rely on computer graphics, which over three decades have become an index of “illusion and artifice” (Gaboury 2021, 9) to illustrate a wide variety of abstractions ranging from the fantastical to the technical: geometric shapes, isometric illustrations, infographics, icons, and diagrams. *Computational abstraction* is the register dominated by minimalist CGI renderings with a color palette centered around glowing blue accents on light grey or black backgrounds. At its most fantastical, this register follows a long tradition of imagining cyberspace and virtual worlds through glowing grids and neon contours segmenting a non-descript black void (Chun 2006, 41), while also rehashing metaphorical representations of big data as an atmospheric force, an information flood, or a resource to be extracted (Puschmann and Burgess 2014; Wyatt 2021). Common elements of this visual register include circuit boards, network diagrams, glittering clouds of dots, concentric rings, hexagonal tiles, mathematical curves, waves of data points, and streaks of lightning [Fig. 5], which are often combined into evocative illustrations used as backgrounds for slideshows, banners or entire webpages. ByteDance, for example, illustrates the features of its algorithm (without specifying which one) through concentric rings made of shape-shifting blue curves, while CloudWalk’s “core technologies” are represented by wireframe illustrations of a human face, a globe, and a brain, all made of networked nodes. Megvii’s AIoT Smart Public Safety Management Solution is summarized by a single image of a glowing lock containing a blue circuit board pattern over a background of flowing data lines, and DJI visualizes their drones’ sensing capabilities as concentric waves or cones of light that allow them to sense their environs as grids or point clouds.

**Fig. 6:**
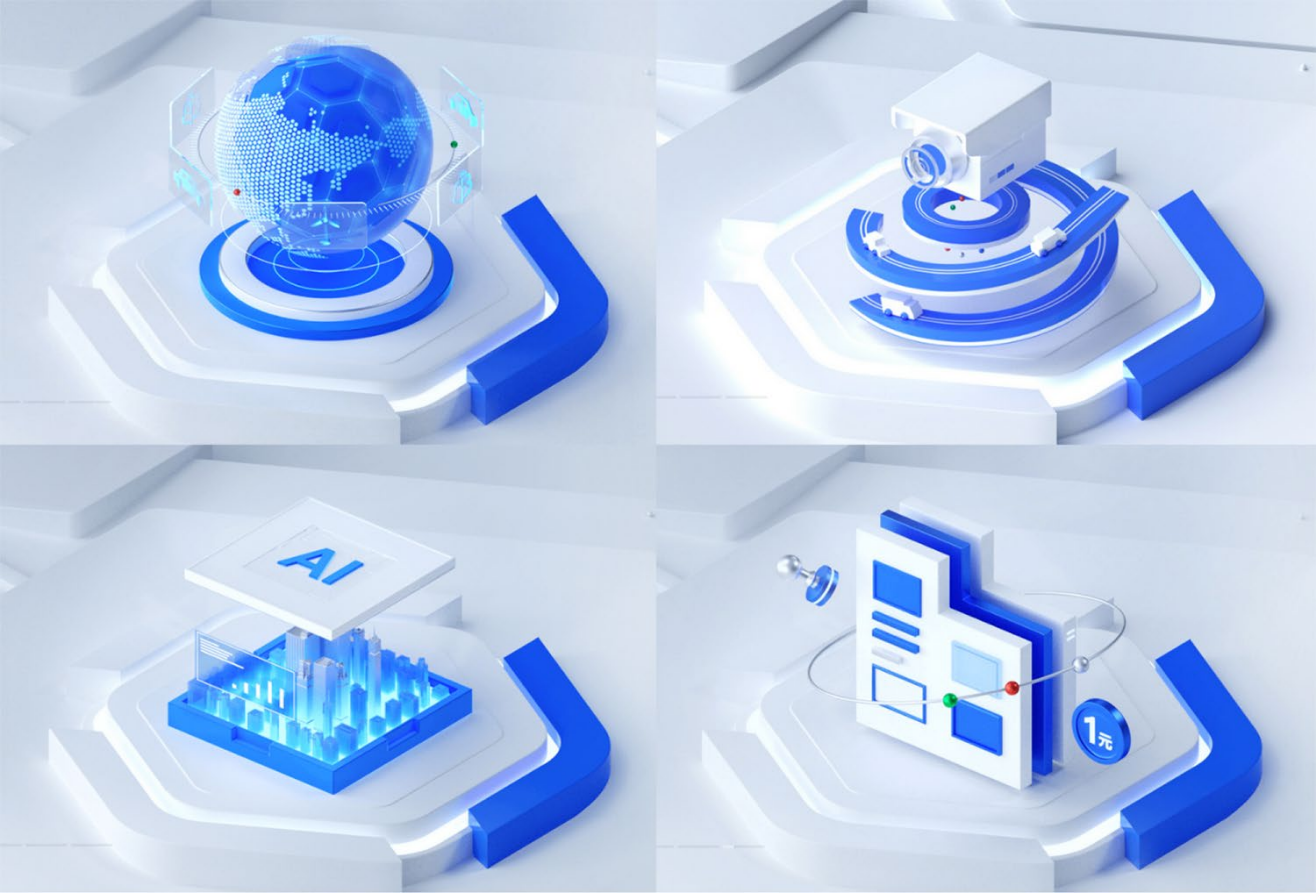
Four isometric illustrations used as backgrounds in the homepage slideshow of the Baidu Cloud website to accompany information about an AI carbon neutrality white paper, a smart content production product, a data security solution, and a year-end special offer

Computational abstraction is also useful to simplify complex systems into consumer-friendly products. Stylized isometric illustrations condense the workings of machine vision into compositions of familiar elements: screens, microprocessors, smartphones, file icons, spinning gears, shields, boxes, OS windows, e-mail icons, fingerprints, clouds, buttons, sliders, checkmarks, and so on. These eye-catching contraptions, which are often animated or responsive, are used as header images for individual product pages, showcasing how the underlying technology works in a dynamic and endearing way. Some of Alibaba Cloud’s machine vision products, for instance, are represented by a satellite circling the globe and beaming down data (AI Earth), an iris-like device scanning an image file icon (Image Recognition), and a robotic eye identifying a stylized person (Face Recognition). Very similar isometric illustrations recur in several of the analyzed websites, particularly the ones of companies selling software solutions [Fig. 6]. Smaller icons designed in the same minimalist geometric style are used to highlight core technologies or recurring features (accuracy, speed, convenience, competitiveness) that are commonly attributed to data analytics (Beer 2017). Larger combinations of these elements connected by arrows, dashed lines or conveyor belts are used as infographics explaining the steps of technical processes like face recognition or threat detection in a simplified manner. Through fluid animations and frictionless connections between parts, this sort of computational abstraction supports a modular view of machine vision (Moss and Schüür 2018) as a toolbox of components that can be “bolted on” one another to construct personalized solutions.

Near the other end of the abstraction spectrum, closer to the technical than to the fantastical, are the stack diagrams used to explain how different components of complex machine vision systems are layered. This sort of diagrams, styled after the OSI (Open Systems Interconnection) model, describe the structure of a system by dividing it into layers each containing modular elements, each layer connected to the one above and below it, with information moving upwards from physical devices and data infrastructures to high-level computational processes and user-friendly applications. Megvii’s Pangu AIoT enterprise platform, for example, is illustrated by a stack diagram grounded on a hardware layer of cameras which feed footage into a middle layer of servers, storage units and computing terminals. These then communicate with a software platform which bundles raw metrics (face capture, vehicle trajectory, smoking, mask wearing) into more structured solutions (access control, people management, behavioral analysis, attribute recognition). Individual hardware and software components are often depicted with isometric illustrations or minimalist icons, and the connections between them are simplified into linear flows and cascades of data. All these representations, from evocative elemental fantasies of data flows and algorithms to streamlined diagrams of services and systems, conjure an atmosphere of “enchanted determinism” (Campolo and Crawford 2020) in which “magical mystery and technical mastery curiously work together” (p. 4) and abstraction clarifies complexity. In the terminology developed by Jancsary et al. (2017), this visual register has an “abstract coding orientation” (p. 99); the observer floats through or above 3D renders of networked brains and circuit boards and is invited to inspect the transparency of computational processes from the inside.

### Human–machine coordination

If computational abstraction is the register of disembodied and stylized technical processes, *human–machine coordination* anchors them to the interaction of embodied actors. In terms of advertising industry products, this register serves the purpose of presenting inspirational encounters between users and machine vision technologies with a range that again spans from the highly metaphorical to the ordinary. Common imagery includes stereotypical portrayals of robots and cyborgs, effortless melds of human bodies and machinic components, analogies between biological organs (eyes, brains) and technological elements (camera lenses, networks), and moments of mirrored or mediated touch between people and technologies. When settling on the name for this register, we have adopted a buzzword emerging from the data, mitigating its markedly gendered form: one of the companies in our sample, CloudWalk, presents itself as “the world’s leading man–machine coordination platform” and dedicates a substantial section of its website to this concept. On the Core Technologies page, a large image depicts the shiny android head looking at augmented reality visualizations of its own designs; a human finger seems to be touching and manipulating them. “Man–machine coordination”, CloudWalk explains, will be key in constructing “a future city with ambient intelligence”. Several infographics (drawing heavily on computational abstraction) further specify features and breakthroughs in man–machine interaction, concluding that CloudWalk’s open platform allows users, manufacturers, clients and services to achieve “co-fusion” and “co-creation”. In practical terms, the company showcases their partnership with the city of Guangzhou for the creation of its “first AI library”, which consists of a face recognition system for book loans, personalized AR services, and thermal analysis of visitor flows. Rather than showcasing the library, the header image depicts the hands of a man in a suit touching glowing icons projected over a tablet.

**Fig. 7:**
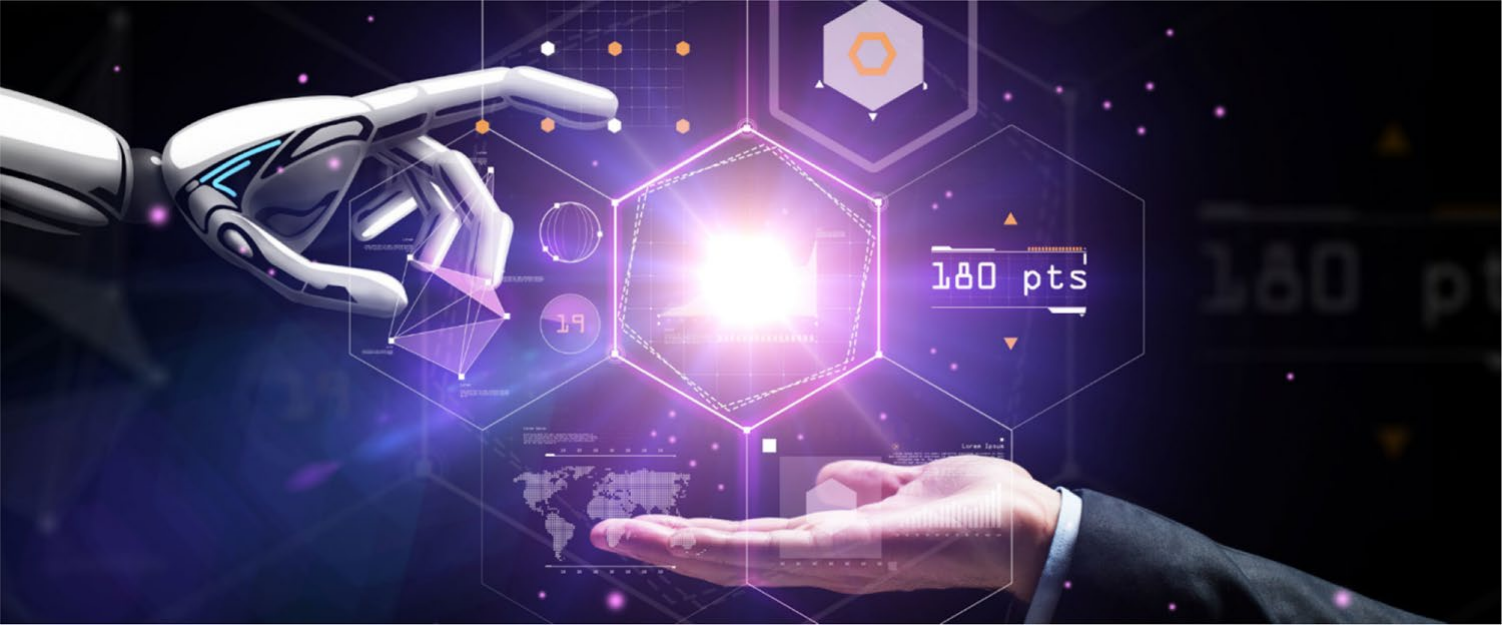
Idealized human–machine coordination on the ByteDance Volcano Engine website

At its most metaphorical, this register relies on both an entrenched organicist imagination of networked infrastructures (Picon 2018, 7–8) as well as the tropes around sentient AI and robotics circulating on popular media (Goode 2018). This combination results in a homogeneous repertoire of composite illustrations, often used to accompany text or set the tone for a webpage, in which humans and machines encounter one another in a set number of ways. Human–machine coordination is also strikingly distant from many of the products it is used to promote, functioning more as an idealized counterweight to vague technical specifications. For example, ByteDance’s Renxiang Renti product [literally, ‘Human Image Human Body’] is presented as a solution combining various forms of image analytics and processing (face recognition, body tracking, GAN-powered filters, etc.) geared towards entertainment, marketing, image optimization, and security–in short, it is a bundle of machine vision applications with no clear use cases. Moreover, its product page does not contain any actual use scenario but instead relies on three illustrations: a close-up of a human eye with rings around its iris and data visualizations in the background; a series of identical robotic hands tapping on interfaces; a Caucasian woman manipulating the hologram of a man in front of abstracted data visualizations. The sections detailing the main product scenarios are similarly steeped in the aesthetics of human–machine coordination – “Interactive Marketing”, for instance, is summarized by a 3D rendering of an android’s hand extending its fingertip to touch a glowing hexagonal pattern containing various data visualization holograms hovering over a human hand [Fig. 7]. Visitors are asked to imagine how this highly metaphorical illustration translates into the “festivals, exhibitions, marketing and other event scenarios” in which this product can be used, while recurring references to reducing costs and enhancing efficiency point to the main value propositions coded by this register.

**Fig. 8:**
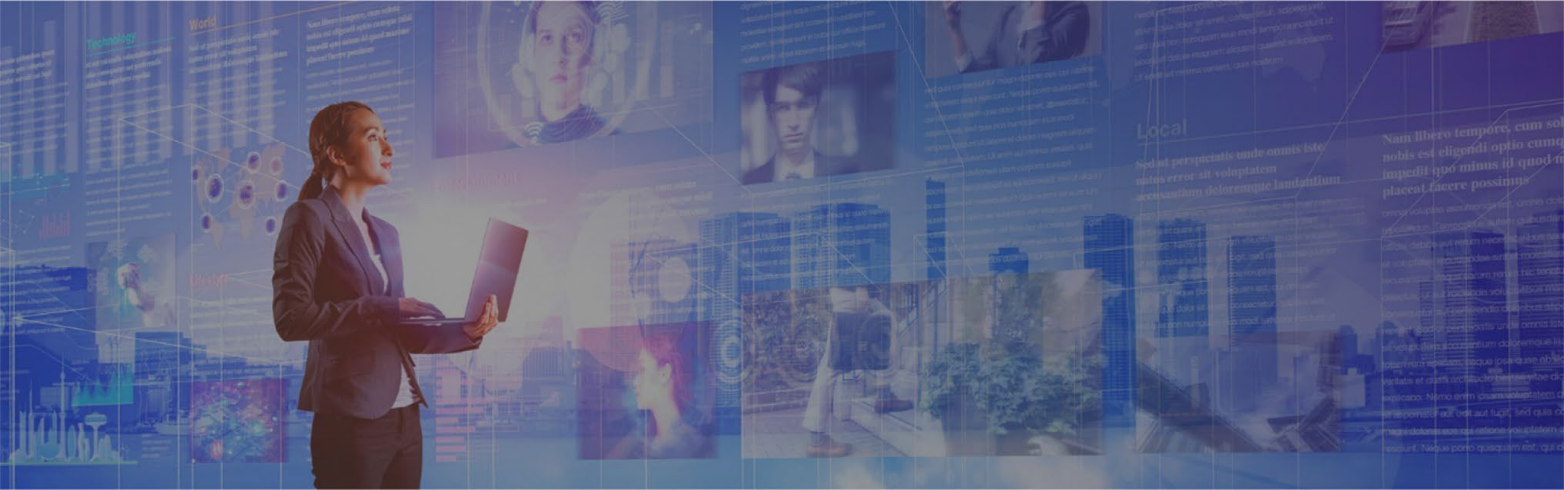
More mundane representation of human–machine coordination on the Huawei website

The metaphorical side of this register represents embodied encounters and coordinated interactions between humans and superhuman technologies, which are “portrayed as *exceeding* human intelligence or performance, as forms of ‘genius’ or even alien cognition” (Campolo and Crawford 2020, 13). Anthropomorphic robots and androids from non-descript futuristic settings abound, often with a focus on their heads (and computational brains), eyes (as cameras, lenses, sensors) and arms, hands, fingers (interacting with screens or other interfaces). The analogy between human brains and thinking machines has a long history in artificial intelligence imaginaries (Gere 2004) and in neuroscientific aesthetics (Grieser 2017), and even the yearning touch between a human and a robotic hand has been identified as a near-religious industry trope (Singler 2020). On the more mundane side of the human–machine coordination register, the human body takes center stage: SenseTime’s SenseFoundry smart city products are illustrated by the hand of a man in business suit that extends to touch and manipulate the hologram of a skyline; Yitu’s Healthcare Total Solution platform is summarized by a Caucasian medical professional looking intently at some circular data patterns floating in the air; on most of the websites we analyzed, photos of professionals interacting with more or less stylized technologies emphasize this need to reach out and engage [Fig. 8]. This register establishes the need for humans to participate in the integration with superhuman technologies (Moss and Schüür 2018, 278) by positioning the visitor as an external observer of encounters with science-fictional embodiments of artificial intelligence. Human–machine coordination emphasizes this moment of encounter by focusing on touch and co-presence, adopting a “sensory coding orientation” (Jancsary et al. 2017, 98) that enhances the affective impact of photomontages through glowing edges, luminous globes, lens flares, and saturated colors.

### Smooth everyday

**Fig. 9:**
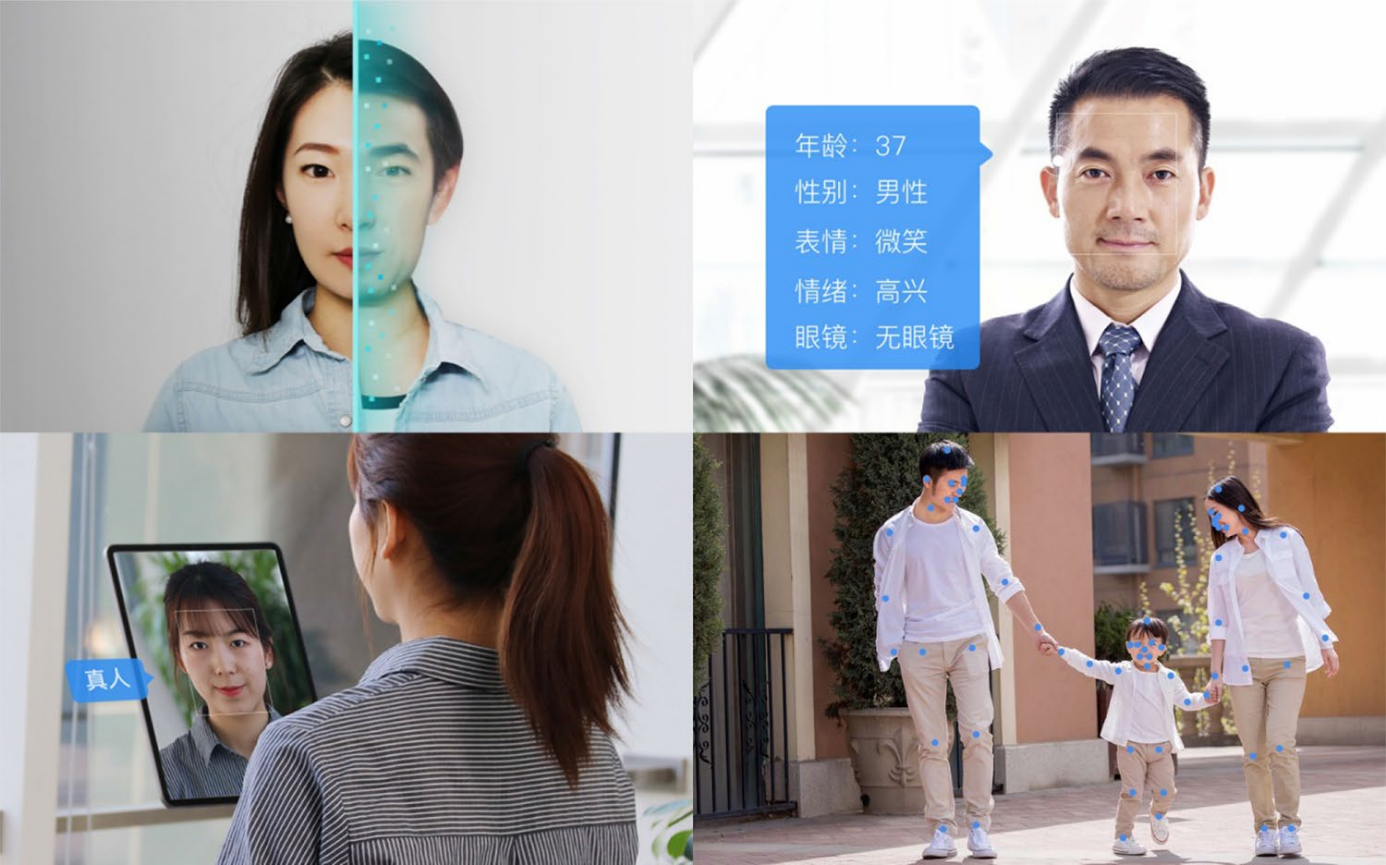
Smooth everyday interactions between people and machine vision technologies on Baidu’s Face Recognition product page

Whereas human–machine coordination imagines the integration of people and machine vision technologies in terms of a highly stylized and stereotyped encounter, the visual register we call *smooth everyday* depicts the real-world adoption and domestication of machine vision. This idealized representation of everyday life relies on photographs of people and settings in which machine vision operates, and recurs in pages and sections illustrating different product scenarios or use cases [Fig. 9]. The photographs of people and settings are often clearly sourced from stock photo libraries, contributing a degree of genericity, timelessness, low modality, and simplification (Machin 2004) to the web page on which they are used. For example, Baidu Cloud illustrates different product Scenarios through scenes of everyday life: pedestrians walking on a city street, women shopping in a mall, two smiling girls taking a selfie. In this visual register, machine vision technologies become almost invisible: they are either assumed to operate in the background of a scene, or their activity is illustrated by translucent light blue overlays – bounding boxes, feature tracking points, data labels – which index specific functions like face recognition or object tracking. These depictions of everyday life share several common aesthetic choices: bright lighting, clean environments, pastel colors, blurred backgrounds, and happy or satisfied users. We call this everyday ‘smooth’ because the adjective encompasses several features emphasized by this register such as convenience, simplicity, reliability, effortlessness, and ubiquity. A video promoting Dahua’s access control point follows a Caucasian employee who conveniently and effortlessly unlocks the door of his with a face scan, even when his hands are busy holding boxes. Alibaba Cloud’s Image Recognition capabilities are demonstrated with photos of objects like laptop computers, coffee cups or snowboards, that the product can reliably identify and categorize. And a SenseTime video showcases how its driver fatigue monitoring product can reliably warn a father who nearly dozes off at the wheel of his family car.

Machine vision makes everyday life smoother – this visual register reinforces a simple message by depicting a very specific range of people and environments with no room for imperfections, failure or doubt. The use of stock images, choreographed scenes and scripted testimonials results in a sanitized representation of everyday life with an overt normative coding of gender, ethnicity and class. Throughout all the websites we analyzed, men and women are portrayed in markedly different ways: men in professional settings, women in domestic ones; men access buildings or drive vehicles, women shop or enjoy leisure time. For example, the Smart Beautification Effects offered by ByteDance’s Volcano Engine (stickers, filters and special effects) are all illustrated by photos of young women applying makeup, taking selfies, or doing heart gestures while on a video call. The people populating the smooth everyday of machine vision are either Asian or Caucasian, with very few examples of other ethnicities – often, Caucasian models are more used in the international version of a company’s website or to demonstrate global reach and partnerships. For instance, Alibaba Cloud’s Face Recognition product is illustrated by stock photos of both Asian and Caucasian women, couples and families; all the protagonists are smiling, pointing to the camera, playing music, or happily hanging out while light blue dots and labels overlaid on their faces exemplify how the technology can detect four key attributes: “gender (male/female), age, expression (laughing/not smiling), and glasses (worn/not worn).” The Scenario section for the same product exemplifies how class is imagined in relation to machine vision: a well-dressed businessman embraces biometric verification to keep his identity credentials safe, while a construction worker is monitored by an object recognition system to ensure he is wearing a helmet while operating heavy machinery.

**Fig. 10:**
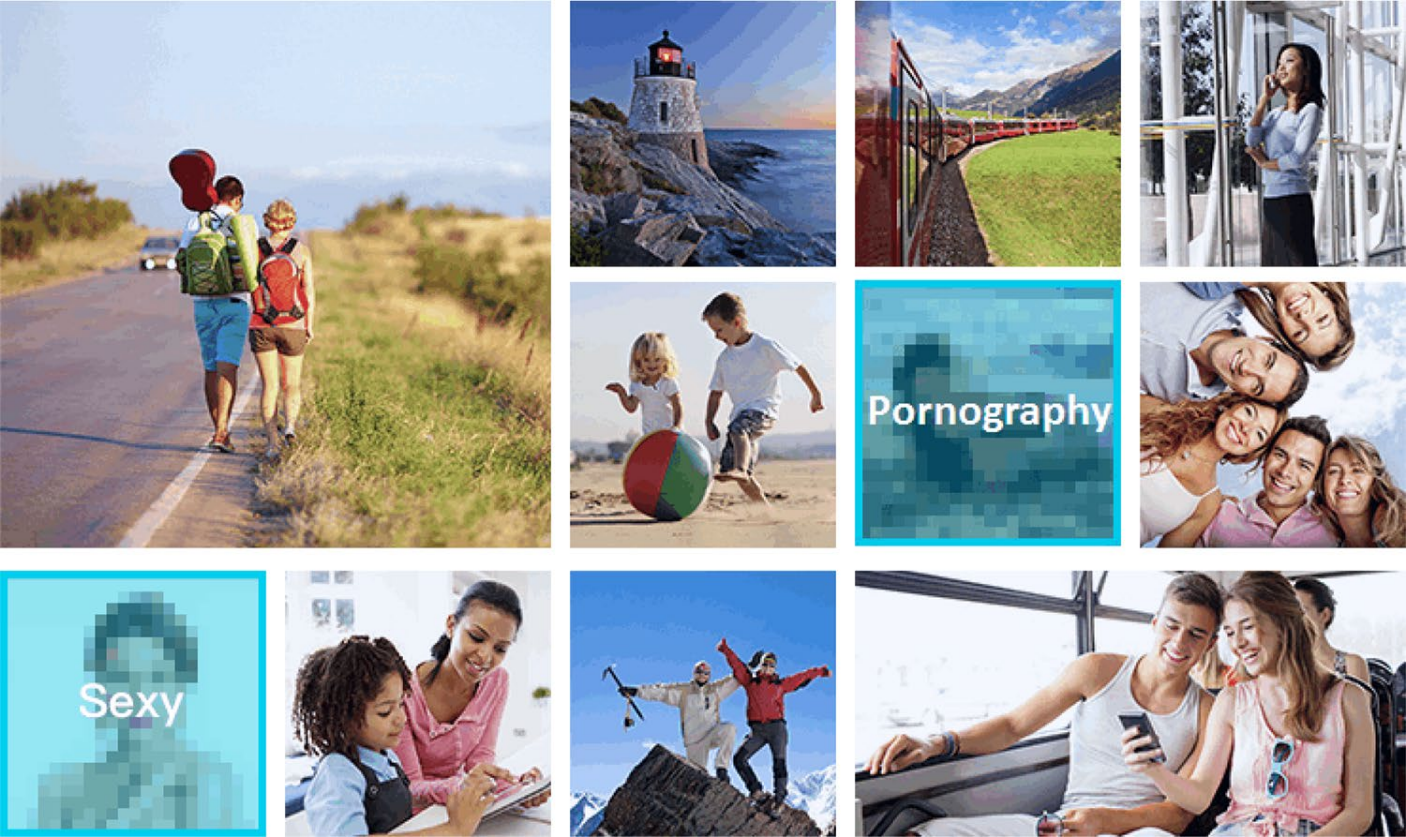
Huawei’s Content Moderation product illustrated as identifying, pixelating and labeling ‘non-compliant’ images among a variety of everyday life scenes

With its bright, minimalist and clean aesthetics, this visual register seeks to represent machine vision as effortlessly integrated in everyday human sociality – people go about their life in naturalistic settings, and technologies are either invisible or peripheral parts of the picture. And yet, many of the websites we analyzed evidenced a striking discontinuity between the people populating this smooth everyday and the actual customers addressed by these companies: with the exception of DJI and Huawei, which also target private consumers, the websites of Chinese machine vision companies are mainly directed at industrial, government and enterprise customers. This sanitized and normative vision of a smooth everyday is itself a product that machine vision technologies promise to bring about for the customer’s own employees, citizens or consumers. Lastly, this visual register actively demarcates the rougher parts of everyday life that machine vision can excise and keep under control: as the page of Huawei’s Content Moderation product illustrates, “non-compliant” content such as pornography and advertisement – with its potential for legal and business risks – is reliably identified and neutralized, preserving the harmony of a bright and clean living [Fig. 10]. The smooth everyday visual register relies on what Jancsary and coauthors define as a “naturalistic coding orientation” (2017, 98). People and technologies are depicted in easily interpretable contexts of activity, dynamic angles and direct eye contact invite viewers into the scenes, and a sense of familiarity, closeness or even intimacy with these visuals fosters an atmosphere of trust and accountability (p. 105). Machine vision smoothens everyday life by almost disappearing into a glowing blue overlay.

### Dashboard realism

**Fig. 11:**
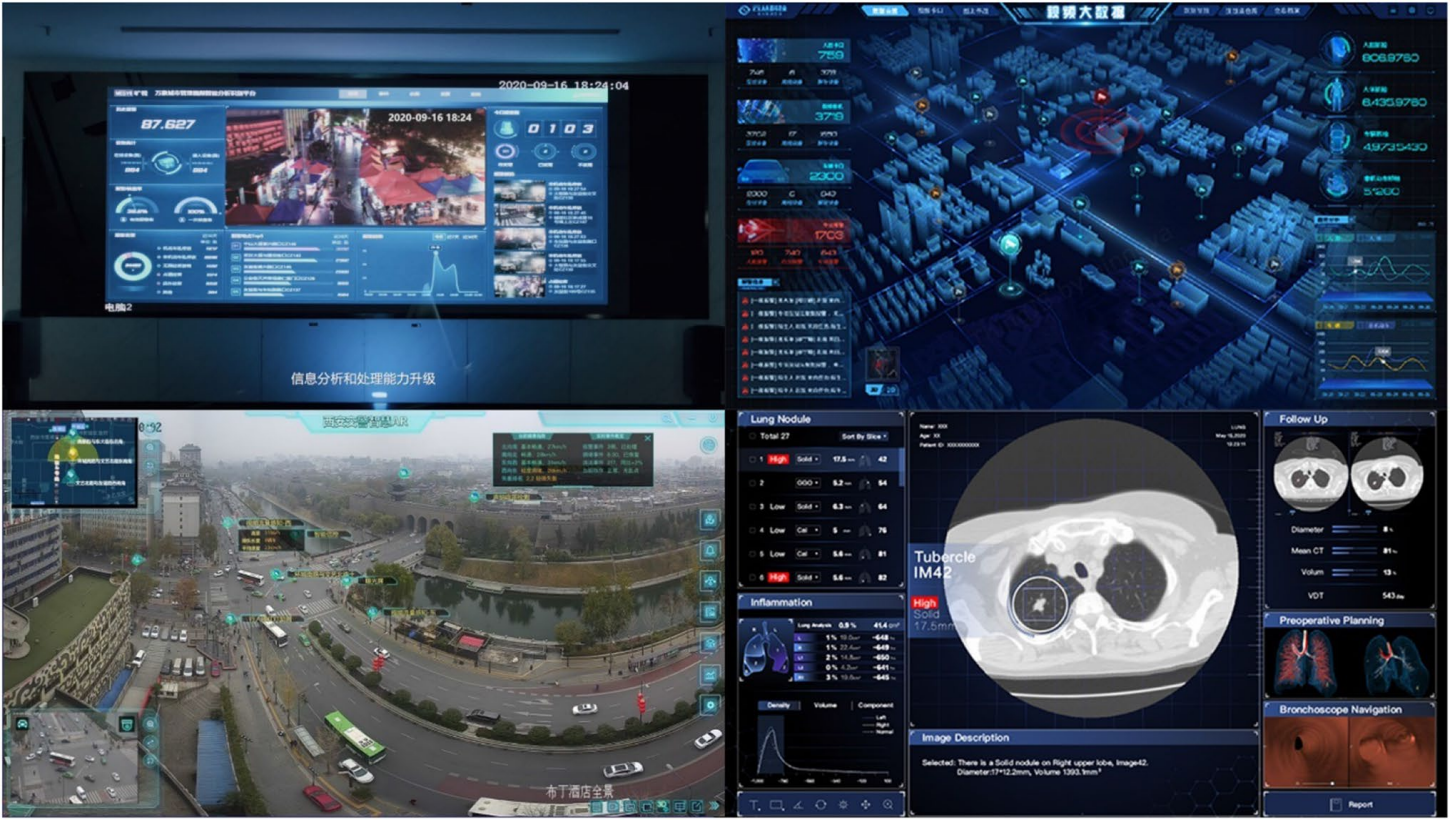
Photos and screenshots of dashboards used to illustrate machine vision products. From left to right, top to bottom: Megvii’s Wanxiang smart city governance platform, CloudWalk’s Video Big Data System, Hikvision’s Intelligent Traffic Management solution, and SenseTime’s SenseLung

While naturalistic depictions of ordinary activities and settings idealize the smooth everyday offered by machine vision, a distinct visual register that we call *dashboard realism* represents the use of these technologies by centering a key interface: the dashboard. The term dashboard – originally referring to control and instrument panels in vehicles – has become adopted as a metaphor in informatics and design to describe various kinds of visual interfaces for the structured display of critical data. With the rise of distributed computation and data-driven governance, dashboards have proliferated across industries and sectors, shaping how the world is interpreted through “epistemological pastiches” of metrics and variables (Mattern 2015). As evidenced by all three previous registers, machine vision is difficult to represent visually, both because of the variety of technologies and products it encompasses, and because its computational processes are largely hidden from the user. Dashboards offer a convenient solution to this problem. Most of the company websites in our sample utilize the recognizable visual features of software dashboards to illustrate how machine vision works and how its users can interact with products [Fig. 11]. These features include God’s eye views of data (from surveillance cameras, drones, satellites), augmented overlays (bounding boxes, colored blobs, labels), and a wide variety of data visualizations (charts, maps, 3D renderings). For example, the Dahua Security Software is marketed as facilitating the human viewing of surveillance camera feeds through various “management modules” targeting people or vehicles and an AI-powered search function capable of tracking faces or license plates across video footage. All these functions are accessible from a user-friendly interface compatible with both desktop and mobile devices, as illustrated by screenshots of actual software dashboards collating different kinds of visualizations and data feeds.

Often showcased on the wall-mounted screens and operator desks of control centers, dashboards have become part of the smart city imaginary, exemplifying how urban space can be made “legible and controllable” (Sadowski 2021, 2) through data and computation. The City Visual Intelligence Engine, one of Alibaba Cloud’s flagship products, is described as a “distributed computing and storage platform” that can open and connect the visual resources scattered in various units of the city, allowing images and videos to help the city in its thinking, decision-making, and operation, thereby improving the overall efficiency.

By collating data and distilling actionable metrics through customer-friendly dashboards, these products promise more efficient governance and connect machine vision to the realization of smart cities. In some cases, companies choose to utilize screenshots and photographs of actual product dashboards on their websites. A news update about SenseTime’s involvement in the digitalization of the Shanghai West Bund area is illustrated by photos of a control room featuring multiple desks and a massive video wall on which a live view of the Huangpu River skyline is overlaid with various windows containing metrics and warnings. The description reinforces the dashboard’s message: “Over 98% of anomalies can be resolved within 20 min, providing high-value comprehensive management services to the customer and significantly enhancing resident and visitor safety experience.” In its Partnerships section, Hikvision’s website details how Miniso retail stores in Poland have installed a system of people-counting cameras, fisheye cameras and network video recorders coordinated through the HikCentral software platform capable of generating visitor heatmaps and purchase statistics, helping the store to manage product allocation. Dashboard realism does not just rely on the technical features of dashboards to visualize machine vision, but also mobilizes the “promissory visions” of data analytics (Beer 2017) to promote products in terms of speed, accuracy and efficiency, directly addressing their contribution to the management and governance of private and public space.

**Fig. 12:**
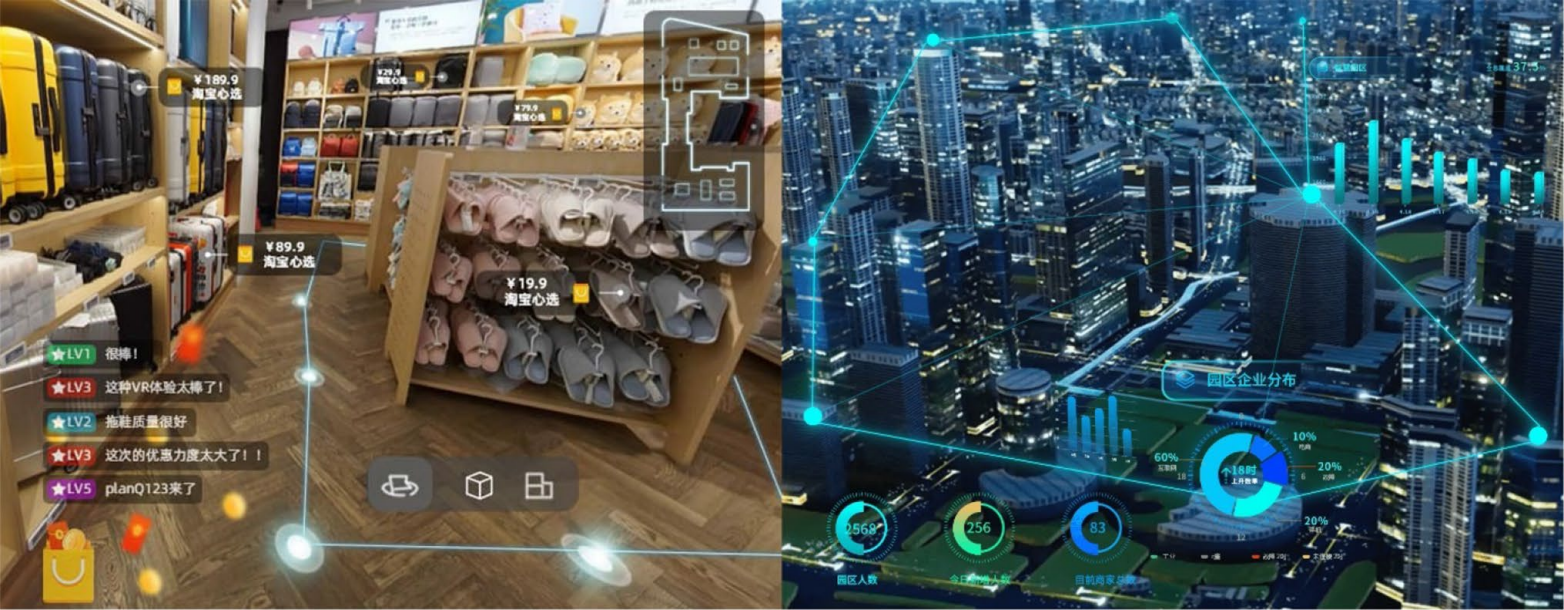
Dashboard elements used in interface mockups illustrating Alibaba’s Holographic Space product

Dashboard realism is not limited to the use of dashboard screenshots or photographs: the structuring logic of dashboards becomes an aesthetic repertoire that machine vision companies can use to explain how their products make the world legible and manageable by humans. The design decisions behind data visualization structure specific ways of “knowing, seeing, enacting and governing populations” (Ratner and Ruppert 2019, 3), and the resulting interfaces can be translated to other contexts as interpretive anchors. The Holographic Space solution offered by Alibaba Cloud, for example, illustrates augmented reality scenarios for domestic entertainment and retail navigation by overlaying dashboard elements on first-person footage of a living room and a store: viewers can easily interpret how the navigation paths, price popups, information labels and expandable dots promise to structure their product experience [Fig. 12]. In some cases, the dashboards are only mockups of future products. On the SenseCore product page, SenseTime showcases its autonomous driving solution through a 3D rendering of a car’s dashboard equipped with a large screen displaying a 3D map of city blocks and information about the vehicle’s system status. The software dashboard is reunited with its hardware predecessor, and the CGI rendering of machine vision promises a future in which mobility can rely on “calculable visibility” (Carlsson 2022, 18) to make streets and city blocks interpretable by cars. Regardless of the sort of dashboards represented by the examples discussed above, this visual register is the only one in which an actual component of machine vision products and systems is depicted in detail. Viewers are assigned a second-person perspective, looking at dashboards from the insider point of view of an ideal user, and the coding orientation of these visuals is largely technological – that is, one “mostly concerned with counting, weighing, and measuring, or providing useful ‘blueprints’ of social reality” (Jancsary et al. 2017, 98–99). Dashboard realism foregrounds a specific genre of user interface as a synecdoche for machine vision as a whole, reinforcing a view of how these technologies can make the world legible as a bundle of metrics and data points.

## Conclusion: Beyond the blue

Machine vision is a key domain of applied artificial intelligence. Like many other novel articulations of technology and society, machine vision is defined, developed and explained by different actors through the work of imagination, resulting in the “collectively held, institutionally stabilized, and publicly performed visions of desirable futures” (Jasanoff 2015, 4) that STS scholars call *sociotechnical imaginaries*. In light of the market size and global scale of China’s AI industry, we asked two questions: What kind of sociotechnical imaginary is articulated by the Chinese machine vision industry? And how do different visual registers contribute to this articulation? To answer these questions, we analyzed the websites of twelve leading industry actors, comparing their use of visual content in search of emerging patterns and significant differences. Our first finding is that Chinese machine vision companies do in fact articulate a cohesive sociotechnical imaginary through consistent aesthetics and design decisions. This imaginary, which we characterize as global, competitive and blue-tinged, emphasizes their international reach and ambitions, their techno-scientific competitiveness, and their shared framing of machine vision as a vague amalgamation of artificial intelligence, machine learning and algorithms. This is significative because it suggests that Chinese companies promote a singular corporate vision that strongly influences how a broader machine vision imaginary is institutionalized, becoming dominant in public discourse and shaping future investment, policies and practices. Tech industries are central actors in the articulation of sociotechnical imaginaries, and in the case of China this is compounded by the pivotal role that these companies play for authoritarian capitalist governance by providing surveillance and security infrastructures (Huang and Tsai 2022, 26). China’s national policies already embrace this vision of artificial intelligence as a technological solution for social problems (Bareis and Katzenbach 2021, 856), so its extension to other global contexts and the possibility of alternative imaginaries emerging and successfully contesting it remain open questions.

**Fig. 13:**
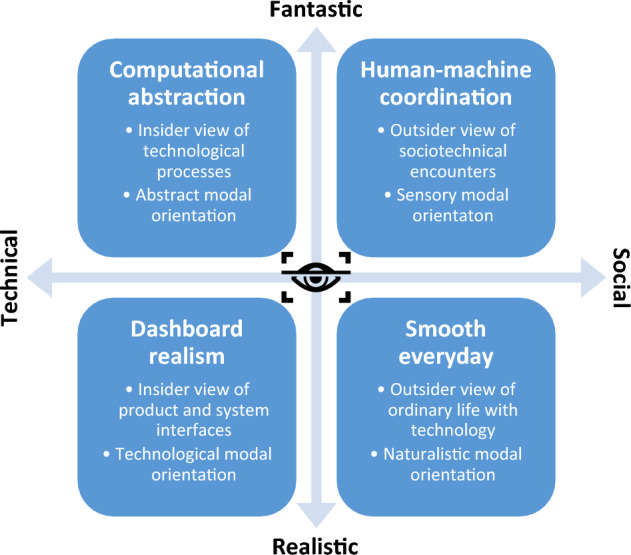
The four visual registers contributing to the sociotechnical imaginary of machine vision

Our second finding is that Chinese machine vision companies articulate a cohesive sociotechnical imaginary through four visual registers defined by specific aesthetics, embodied positions, and modal orientations [Fig. 13]. These registers help companies illustrate a wide range of technologies that are notoriously difficult to explain to a non-specialized audience. Through the visual registers of *computational abstraction*, *human–machine coordination*, *smooth everyday*, and *dashboard realism*, website visitors are encouraged to imagine machine vision at different scales: the abstracted insides of chips and servers; the sensuous moments of encounter between humans and more-than-human technologies; the convenient daily interactions with automated systems; the efficient use of interfaces for management and governance. These registers can be seen as a continuum: for example, adding the social to computational abstraction leads to visions of human–machine coordination, and elements of dashboard realism contribute to depictions of smooth everyday scenes. This representational loop encompasses the social and the technical, the realistic and the fantastic; while the four registers can be applied quite distinctively, they are also not mutually exclusive and are often combined to achieve specific representational effects, particularly in time-based media such as the promotional videos described in the introduction . From our analysis of Chinese corporate websites, we conclude that the sociotechnical imaginary articulated by these four visual registers hides the functioning of machine vision technologies behind the abstract and the fantastic, and depicts their use through narrow representations of everyday life and actual use. Our study has obvious limitations: a narrow temporal and geographical scope (November 2021 – June 2022, China); a sampling that privileges top companies, missing alternative imaginaries emerging from less visible actors; and a focus on websites that ignores other key sites of articulation (advertisement, tech fairs, internal corporate communications, etc.). Future studies of machine vision imaginaries could move ‘beyond the blue’ and explore how they change in time, conduct cross-national comparisons, or complement our findings with quantitative or fieldwork-based research.

## Data Availability

The datasets generated during and analysed during the current study are available from the corresponding author on reasonable request.

## Appendix 1: analyzed websites

Alibaba Cloud: www.alibabacloud.com

Baidu Cloud: intl.cloud.baidu.com, cloud.baidu.com.

Bytedance’s Volcano Engine: www.volcengine.com

CloudWalk: www.cloudwalk.com/en, www.cloudwalk.com

Dahua: www.dahuasecurity.com/ceen, www.dahuatech.com

DJI: www.dji.com

Hikvision: www.hikvision.com/en, www.hikvision.com/cn

Huawei Enterprise: e.huawei.com/en.

Megvii: en.megvii.com, megvii.com.

SenseTime: www.sensetime.com/en, www.sensetime.com/cn

Tencent Cloud: intl.cloud.tencent.com, cloud.tencent.com.

Yitu: www.yitutech.com/en, www.yitutech.com/cn

## Appendix 2: Industry rankings

China Money Network (2018) *China AI top 50*. https://www.chinamoneynetwork.com/china-ai-top-50-2018

Chinese Academy of Science (2020). *2019 nian rengong zhineng fazhan baipishu [2019 AI development white paper*. http://pdf.dfcfw.com/pdf/H3_AP202001171374280695_1.pdf

Value Today (2020). *Top technology companies in China by market cap*. https://www.value.today/top-companies/top-technology-companies-china

Australia Strategic Policy Institute (2021). *Mapping China’s tech giants.* https://chinatechmap.aspi.org.au/

eNet&Ciweek (2021). *2021 Rengong zhineng fenlei paihang [2021 artificial intelligence category rankings]*. http://www.enet.com.cn/article/2021/0513/A202105131264814.html

## Footnotes

1 Given the large number of references to pages from the official websites of Chinese tech companies, we do not cite individual pages but provide a full list of all the sampled sources in Appendix 1.

2 Machine vision technologies such as face recognition and image analysis play a key role in the unprecedented scale of China's state surveillance apparatus. Chinese authorities have relied on the products developed by machine vision companies to automate the policing of ethnic minorities (Leibold 2019) and to enforce governance measures during the COVID-19 pandemic (Weinstein 2020). Chinese machine vision companies not only enable nationwide monitoring programs but also drive the expansion of surveillance worldwide (Feldstein 2019), leading countries like the U.S., the United Kingdom, Denmark and Taiwan to ban or restrict the import and sale of their products.

3 Here the exception is revealing: one of the few pages not dominated by blue overtones is that of Megvii’s FaceStyle. The main color through which this “comprehensive beauty solution” is illustrated is pink, and its targeted customers and clearly marked as women.

4 We speculate that the ongoing COVID-19 pandemic as well as the U.S.-China trade war have impacted the international business of many of these companies, rendering the international version of their website less useful and hence less frequently updated.
